# The importance of miRNA-630 in human diseases with an especial focus on cancers

**DOI:** 10.1186/s12935-022-02531-z

**Published:** 2022-03-05

**Authors:** Sepideh Kadkhoda, Soudeh Ghafouri-Fard

**Affiliations:** 1 Department of Medical Genetics, School of Medicine, Tehran University of Medical Sciences, Tehran, Iran; 2 Department of Medical Genetics, Shahid Beheshti University of Medical Sciences, Tehran, Iran

**Keywords:** miR-630, Cancer, Marker

## Abstract

miR-630 is encoded by MIR630 gene (NC_000015.10) on 15q24.1. This miRNA is mostly associated with cytokine signaling in immune system. Several neoplastic as well as non-neoplastic conditions have been linked with dysregulation of miR-630. It is an oncogenic miRNA in renal cell carcinoma, multiple myeloma, colorectal cancer, acute lymphoblastic leukemia, ovarian cancer and prostate cancer. On the other hand, it is a putative tumor suppressor miRNA in lung, cervical, breast, thyroid and esophageal tissues. In a number of other tissues, data regarding the role of miR-630 in the carcinogenesis is conflicting. Expression levels of miR-630 can be used as markers for prediction of cancer course. Moreover, miR-630 can influence response to chemoradiotherapy. This miRNA is also involved in the pathoetiology of IgA nephropathy, obstructive sleep apnea, age-related nuclear cataract and vitiligo. In the present review, we discuss the role of miR-630 in these conditions.

## Introduction

MicroRNAs (miRNAs) are small sized transcripts with no capacity of protein coding [1]. miRNAs can bind to coding sequences and miRNA seeds in coding regions are coordinated with regulation/influencing the targets [2–4]. Moreover, various molecular techniques like reporter assays confirms that miRNA seeds in coding region can be functional and influence the protein synthesis [5, 6]. Mature miRNAs have sizes about 22 nucleotides and are made from their precursors in a multistep process being catalyzed by Drosha and Dicer RNase III proteins. These transcripts serve as guide molecules for suppression of expression of target RNAs. Since they can target the majority of protein-coding RNAs, miRNAs partake in virtually total physiological and pathological events. miRNA synthesis is tightly controlled in terms of time and location. Not surprisingly, dysregulation of expression of miRNAs is linked with the pathoetiology of several human diseases, above all being cancer [7]. miR-630 is an example of these transcripts which is encoded by *MIR630* gene (NC_000015.10) on 15q24.1. This miRNA is mostly associated with cytokine signaling in immune system. Moreover, this miRNA has functional interactions with other non-coding RNAs such as long non-coding RNAs and circular RNAs. Thus, it contributes in the construction of a complex interactive network that is implicated in the pathogenesis of human disorders. Several neoplastic as well as non-neoplastic conditions such as IgA nephropathy, obstructive sleep apnea, cataract, vitiligo and heterotopic ossification have been linked with dysregulation of miR-630. In malignant conditions, both high throughput sequencing methods and candidate gene expression analyses have confirmed association between dysregulation of miR-630 and disease progression. Although there are several miRNAs that play important roles in the pathogenesis of disorders, we have selected miR-630 with respect to being targeted by important upstream factors such as long non-coding RNA H19 and also targeting several important genes and participation in key signaling pathway such as AKT, P53, TGFβ-ERK/SP1, JNK/c-Jun, PI3K/AKT and JAK2/STAT3 which contribute in the development of various malignant and non-malignant diseases. Thus, in the present review, we discuss the role of miR-630 in these conditions.

## miR-630 in malignancies

**Fig. 1:**
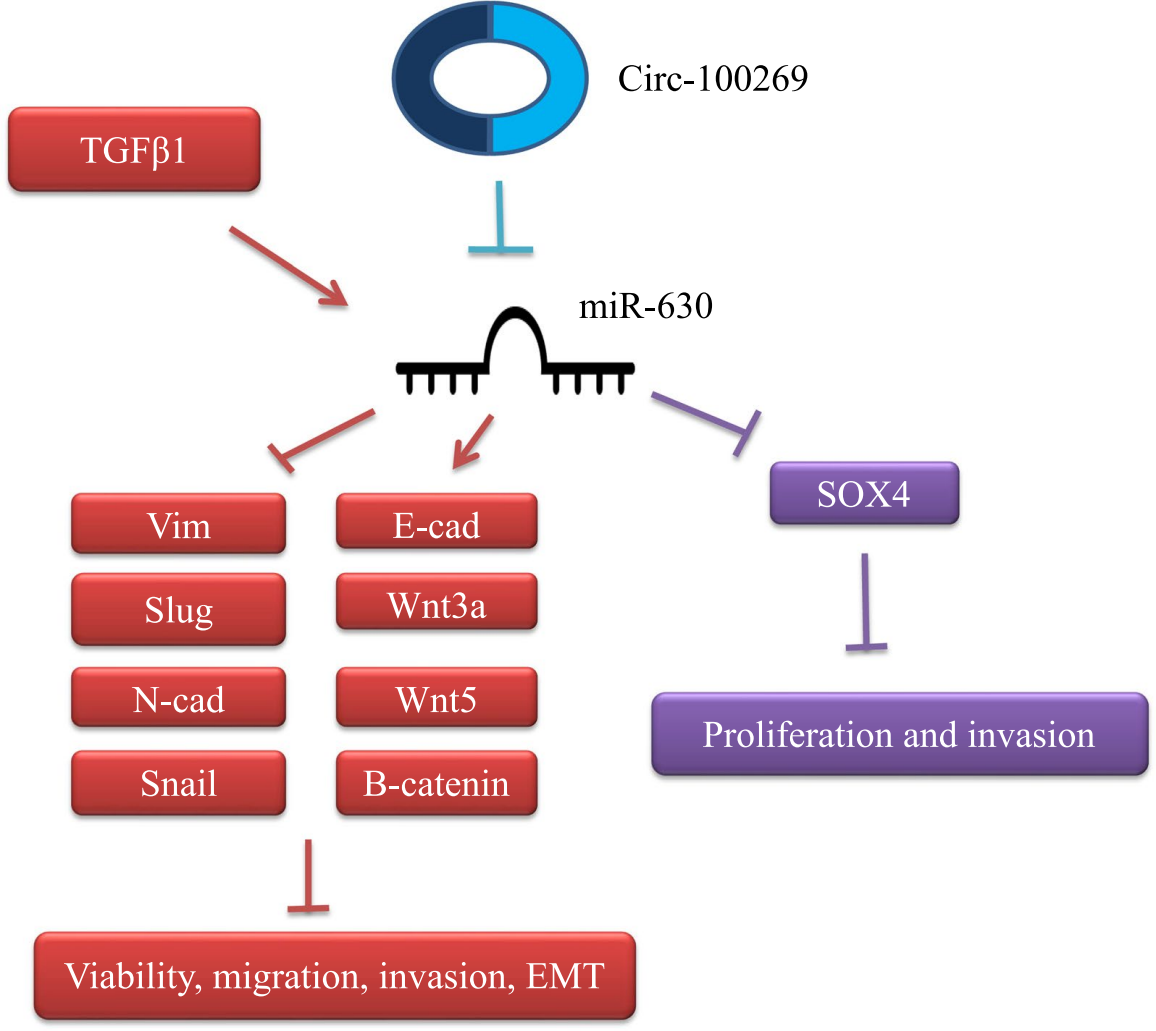
In gastric cancer, circRNA-100269 had a negative correlation with miR-630 and inhibited its expression (blue axis) [19]. In the cells treated with TGF-β, miR-630 via blocking of vimentin, slug, snail, and N-cadherin and also by induction of β-catenin, E-cadherin, wnt3a, and wnt5a inhibited cell viability, migration, invasion, and EMT (red axis) [21]. Moreover, miR-630 through inhibition of SOX4 could suppress invasion and proliferation of gastric cancer cells (purple axis) [18]

miR-630 has been found to be over-expressed in renal cancer cell lines compared with HK-2 normal renal cells. Inhibition of miR-630 has resulted in suppression of proliferation, migratory aptitude, and invasiveness of 786-O renal cancer cells. Moreover, apoptosis has been induced following miR-630 inhibition, indicating potential of this method for treatment of renal cancer [8]. miR‑630 has also been shown to promote proliferation of HCT116 colorectal cancer cells and inhibit their apoptosis. The impact of miR-630 on apoptosis of HCT116 cells has also been verified through the observed reduction of expressions of p27, BAX (BCL2 Associated X), procaspase‑3 and active caspase‑3. Moreover, miR-630 has enhanced levels of phosphorylated‑AKT and BCL2 (B-Cell CLL/Lymphoma 2). Thus, miR‑630 has an oncogenic role in colorectal cancer through modulation of p27 and AKT pathway [9]. In Jurkat cell line, miR-630 has induced cell proliferation and reduced cell apoptosis through affecting expressions of p53, p21 and BCL2 [10]. Expression of miR-630 has been reported to be increased in epithelial ovarian cancer tissues as compared with normal ovarian tissues. In SKOV3 cells, miR-630 up-regulation has shown pro-proliferative and pro-migratory effects, at least partly through targeting KLF6 (Krüppel-like Factor 6) [11]. Moreover, miR-630 has been shown to affect cell apoptosis and sensitivity of ovarian cancer cells to cisplatin through targeting PTEN (Phosphatase and Tensin Homolog) [12]. In a high throughput study using PCR array and NanoString techniques, miR-630 has been found to be among up-regulated miRNAs in tumoral tissues of young patients compared to normal tissues [13]. In hepatocellular carcinoma (HCC), two different studies have reported contradictory results regarding the role of miR-630. Zhang et al. have shown over-expression of miR-630 in HCC samples and cell lines compared with corresponding controls [14]. miR-630 expression has been found to be significantly elevated at advanced TNM stages [14]. Furthermore, up-regulation of miR-630 in tissue samples of HCC has been associated with elevation in serum levels of AFP (Alpha Fetoprotein), indicating its association with HCC progression [14]. On the other hand, Chen et al. have reported that down-regulation of miR-630 in HCC patients is associated with higher chance of tumor recurrence and shorter survival of patients [15]. Functionally, miR-630 has been shown to attenuate epithelial-mesenchymal transition (EMT) in HCC through targeting Slug [15]. Moreover, TGF-β (Transforming Growth Factor Beta 1)-Erk (Extracellular Signal-Regulated Kinase)/SP1 (Specificity Protein 1) and JNK (Jun N-Terminal Kinase)/c-Jun cascades have been demonstrated to repress transcription of miR-630 via taking the position of transcription factors on promoters. Forced over-expression of miR-630 has reinstated the TGF-β-associated EMT [15]. In non-small cell lung cancer (NSCLC), circMTDH.4 has been shown to regulate expression of AEG-1 (Astrocyte Elevated Gene-1) oncogene through sequestering miR-630 [16]. CircMTDH.4 silencing or miR-630 up-regulation has suppressed resistance of NSCLC cells to chemo/radiotherapy, indicating the importance of circMTDH.4/miR-630/AEG-1 axis in modulation of response of NSCLC cells to these therapeutic options [16]. According to colony formation assays, suppression of circ‐MTDH.4 with sh‐circMTDH.4 or/and miR‐630 up-regulation by miR‐630 mimic significantly increased 5‐FU or cisplatin‐induced cell death in A549 cells [16]. Consistent with this study, miR-630 has been found to be down-regulated in NSCLC tissues and cells [17]. Forced up-regulation of miR-630 could suppress proliferation, migration, and invasiveness of NSCLC cells through targeting LMO3 (LIM Domain Only 3), a gene that encodes a nuclear LIM-only protein [17]. miR-630 acts as up-stream regulator for LMO3 [17]. Restoration of LMO3 significantly reversed the anti-cancerous effects of miR-630 on cell proliferation, migration, and invasion in malignant cells. So, miR-630 inhibited the proliferation, migration, and invasion of NSCLC cells by down-regulation of LMO3 level [17]. Expression of miR-630 has been shown to be decreased in serum of gastric cancer patients compared with controls [18]. Notably, levels of miR-630 have been much lower in those having aggressive tumors [18]. Down-regulation of miR-630 has been correlated with poor prognosis of these patients [18]. The tumor suppressor role of miR-630 in gastric cancer has been further verified through the observed reduction in proliferation ability of SGC-7901 cells following over-expression of this miRNA [18]. Functionally, miR-630 exerts these effects through down-regulating expression of SOX4 (SRY-Box 4) [18]. Conversely, in a study conducted by Zhang et al., expression of miR-630 has been reported to be higher in gastric cancer tissues (intestinal, mixed, and diffuse types) compared to corresponding nearby tissues [19]. The tumor suppressor circular RNA circRNA_100269 has been shown to suppress growth of gastric cancer cells through targeting miR-630 (Fig. 1) [19]. Finally, Feng et al. have reported that miR-630 suppresses EMT, migration and invasive features of gastric cancer cells through regulating FoxM1 (Forkhead Box M1) and decreasing expressions of GTP-Rac1, p-PI3K (Phosphatidylinositol-4,5-Bisphosphate 3-Kinase Catalytic Subunit Alpha), and p-AKT [20]. In the cells treated with TGF-β, miR-630 via blocking of vimentin, slug, snail, and N-cadherin and also by induction of β-catenin, E-cadherin, wnt3a, and wnt5a inhibited cell viability, migration, invasion, and EMT (Fig. 1) [21]. Thus, this miRNA has a role in modulation of canonical Wnt signaling [21].

In nasopharyngeal carcinoma (NPC), miR-630 has been up-regulated in serum samples of patients compared with controls, indicating a biomarker role for this miRNA [22]. On the other hand, its expression has been shown to be down-regulated in NPC samples compared with chronic inflammatory nasopharyngeal epithelium. Functionally, H19 through sponging miR-630 could regulate invasiveness of these cells [23]. Table 1 summarizes the role and expression pattern of miR-630 in cancers.

**Table 1:** miR-630 in cancers (*ANCT* adjacent non-cancerous tissue, *GEO* gene expression omnibus)

Type of cancer	Expression pattern	Samples	Cell lines	Downstream targets	Other related molecules and pathways	Function	Refs.
Renal cell carcinoma (RCC)	Up	–	786-O, ACHN, Caki-1, Caki-2 and HK-2	–	–	Inhibition of miR-630 could suppress tumorigenesis	[8]
Multiple myeloma (MM)	Up	Plasma from 40 MM patients and 20 control subjects	–	–	–	miR-630 was categorized as a differentially expressed miRNA between MM patients and healthy subjects	[24]
Colorectal cancer (CRC)	–	–	HCT116	P27, BAX and caspase-3, p-AKT and BCL2	AKT pathway	Overexpression of miR-630 via inhibiting of P27, BAX and, caspase-3 and enhancing p-AKT and BCL2 could increase cell proliferation and immortality. Suppression of it could have an opposite effect	[9]
Acute lymphoblastic leukemia (ALL)	–	–	Jurkat cells	P53, P21, and BCL2	P53 pathway	Overexpression of miR-630 by suppressing P53 and p21 and via bcl2 enhancement could lead to the growth of cancer cells and inhibition of the apoptosis process	[10]
Ovarian Cancer (OC)	Up	30 ovarian cancer tissues, 30 normal ovarian tissues/mice	SKOV3	KLF6	–	Overexpression of miR-630 could enhance tumorigenesis	[11]
Prostate cancer (PCa)	Up	10 pairs of PCa Tumors and ANCTs from young men	–	–	–	miR-630 up-regulated in young men tumor tissues than their normal adjacent tissues	[13]
Hepatocellular carcinoma (HCC)	Up	42 pairs of tumors and ANCTs	SMMC-7721, Hep3B, HepG2, HCCLM3,LO2 and HL-7702	AFP	–	A direct correlation between AFP level and miR-630 expression could be observed	[14]
	–	–	QSG7701, HL7702, HCCLM3, MHCC97H, MHCC97L, SMMC-7721, HLF, Bel7402, HepG2, Hep3B and Huh7	Slug, E-cadherin, N-cadherin, and vimentin	TGF-β-ERK/SP1 and JNK/c-Jun	miR-630 could diminish cell migration, invasion, and EMT and also afford apoptosis. TGF-β by ERK/SP1 and JNK/c-Jun pathways could inhibit miR-630	[15]
Pancreatic cancer (PC)	–	GEO database	–	NTS, CDH2, GRIA2	PI3K/AKT	miR-630 could promote apoptosis by affecting PI3K/AKT pathway	[25]
Non-small cell lung cancer (NSCLC)	Down	22 pairs of NSCLC tissues and ANCTs	NCI-H23, A549, H157, H1299 and 16HBE	LMO3	–	The expression level of miR-630 could have an opposite correlation with LMO3, and its overexpression could suppress tumorigenesis	[17]
Cervical cancer (CC)	Down	10 pairs of cervical tumors and ANCTs	Caski, Ms751, Hela and Siha	Vimentin, N-cadherin, E-cadherin, Snail, Zeb1 and Zeb2	E6/E7-p53 signaling pathway	E6/E7 by inhibition of P53 could suppress miR-630 that provokes invasion, migration, and EMT of cancer cells	[26]
	Down	Mice	Caski, HeLa, C‐33A, SiHa, SW756, and HcerEpic	YAP1	LncRNA NOC2L‐4.1	NOC2L‐4.1 by regulation of miR‐630/YAP1 axis could promote cell multiplication and migration. NOC2L‐4.1 and YAP1 had an inverse correlation with miR-630	[27]
Breast cancer (BC)	Down	43 pairs of tumor tissues and ANCTs	MDA-MB-231-luc-D3H2LN, MCF-10A, MDA-MB-231, MDA-MB-468, MDA-MB-435S, BT-549, BT-474, SK-BR-3, HCC1937, MCF7, and HEK293T	MTDH	–	Over-expression of miR-630 could prevent cell migration, invasion, colony formation, and pulmonary metastasis	[28]
Gastric cancer (GC)	Down	Blood, tumor tissue and ANCTs from 131 patients/blood sample from 116 healthy persons	SGC-7901	SOX4, p21, p27, MMP2, and MMP9	–	Over-expression of miR-630 and as a result decreased expression of SOX4 could reduce proliferation and invasion of cells	[18]
	Up	167 pairs of tumors and ANCTs	AGS, MKN28, MKN45, BGC823, MGC803, SGC7901, and GES1	–	CircRNA-100269	miR-630 as a target of circRNA-100269 could have a negative correlation with this circular RNA. So, it could reverse the inhibitory effects of circRNA-100269	[19]
	–	–	SGC-7901	FoxM1, Ecadherin, vimentin, GTP-Rac1, p-PI3K, and p-AKT	Ras/PI3K/AKT	miR-630 could cause migration, invasion, and EMT decline	[20]
Nasopharyngeal carcinoma (NPC)	Up	Plasma sample from 55 NPC patients and 45 controls	–	–	–	miR-630 could be considered as a new biomarker in NPC	[22]
	–	–	NP69, CNE2, CNE1, and HONE1	EZH2, E-cadherin	H19	H19 as a sponge for miR-630 could regulate EZH2 and led to cell invasion	[23]
Osteosarcoma	Down	147 pairs of tumor tissues and ANCTs	Saos-2, U2OS, MG63, HOS and hFOB1.19	PSMC2, N-cadherin and vimentin	–	miR-630 as an upstream tumor suppressor factor of PSMC2 could inhibit tumor characteristics	[29]
Papillary thyroid carcinoma (PTC)	Down	47 pairs of PTC tissues and ANCTs/GEO database	Nthy-ori3-1, SW1736, 8505C and TPC-1	Caspase3, caspase9, vimentin, N-cadherin, p-JAK2 and p-STAT3	JAK2/STAT3	Up-regulation of miR-630 could inhibit proliferation, invasion, and migration processes and could cause apoptosis	[30]
Esophageal cancer	Down	Peripheral blood from 58 EC patients and 60 controls	KYSE-150, KYSE-450, ECA109 and HEEC	–	–	miR-630 induction in malignant cells could inhibit migration and invasion	[31]
Esophageal squamous cell carcinoma (ESCC)	Down	44 pairs of tumors and ANCTs/mice	Eca109, EC9706, TE1, Kyse-30, Kyse-70 and HEEC	E-cadherin, β-catenin N-cadherin, slug, and vimentin	–	miR-630 upregulation could suppress proliferation, metastasis, EMT, and invasion of cells	[32]

Expression levels of miR-630 could affect patients' prognosis. In renal cell carcinoma, its expression has been correlated with tumor grade, lymph node metastasis, as well as distant metastasis [33]. In NSCLC, low level of miR-630 and high level of BCL2 have predicted poor outcomes in the patients [34]. In HCC, miR-630 up-regulation has been correlated with advanced stage, micro and macro-vascular invasion in a single study [14], while in another study its down-regulation has been associated with high metastasis probability, incomplete encapsulation, high tumor number, and vascular invasion possibility [15]. In gastric cancer, Zhou et al. [18] and Chu et al. [35] have reported totally contradicted results. Table 2 summarizes the impact of miR-630 expression on patients’ survival in different types of cancers.

**Table 2:** Prognostic role of miR-630 in cancers (*ANCTs* adjacent non-cancerous tissues, *BCLC* Barcelona-Clinic Liver Cancer)

Samples	Kaplan Meier	Multivariate Cox analysis	Refs.
33 renal cell carcinoma (RCC) tissues	–	Up-regulation of miR-630 was correlated with TNM stage class I	[36]
92 RCC tissues	Up-regulation of miR-630 was correlated with the unfavorable prognosis of patients	Up-regulation of miR-630 was correlated with the grade, lymph node metastasis, and distant metastasis	[33]
53 ovarian cancer (OC) tissues	–	High level of miR-630 was correlated with higher stages and lymph node metastasis	[12]
114 tumor tissues of non-small cell lung cancer (NSCLC)	Low level of miR-630 and high level of BCL2 correlated with weak outcomes in the patients	The advanced stage of tumors was associated with low level of miR-630	[34]
42 pairs of hepatocellular carcinoma (HCC) tumors and ANCTs	There was a positive correlation between miR-630 expression and the patient’s prognosis	There was a positive correlation between miR-630 expression and advanced stage (II–III), micro and macro-vascular invasion	[14]
97 HCC patient tissues	Low level of miR-630 was associated with the poor prognosis	There was a correlation between low levels of miR-630 with metastasis probability, incomplete encapsulation, advanced edmondson stage, advanced TNM stage, advanced BCLC stage, high tumor number, and vascular invasion possibility	[15]
46 NSCLC tumor samples	Low level of miR-630 was correlated with a worse prognosis	Low level of miR-630 usually observed in advanced stages (II, III, and IV)	[37]
Tumor tissue from 118 gastric cancer (GC) patients	Low level of miR-630 was associated with the poor prognosis	There was correlation between miR-630 expression with tumor size, lymph node metastasis, depth of invasion, and stage	[18]
236 GC tumors (intestinal and diffuse type)	miR-630 up-regulation was associated with poor overall survival	miR-630 over-expression correlated with invasion, lymph node and distant metastasis, and TNM stage	[35]
147 osteosarcoma tissues	Low level of miR-630 was associated with the worse prognosis	There was a correlation between low miR-630 expression with higher stage and distant metastasis	[29]
Peripheral blood from 58 esophageal cancer patients	Low level of miR-630 was associated with poor prognosis	The levels of miR-630 were associated with disease course, stage, differentiation degree, and metastasis	[31]
44 esophageal squamous cell carcinoma (ESCC) tumors	Low level of miR-630 was associated with the unfavorable prognosis	The levels of miR-630 were associated with tumor range, lymphatic metastasis, and stage	[32]
116 pairs of bladder urothelial carcinoma and ANCTs, 42 normal tissue	High level of miR-630 was associated with the worse prognosis	There was a correlation between high miR-630 level with advanced TNM stage, higher grade and, lymph node metastasis	[38]
206 colorectal cancer (CRC)	High miR-630 expression was associated with poor overall survival	High expression of miR-630 correlated with tumor invasion (T3+T4), lymph node metastasis, distant metastasis, and advanced TNM stage	[39]

**Fig. 2:**
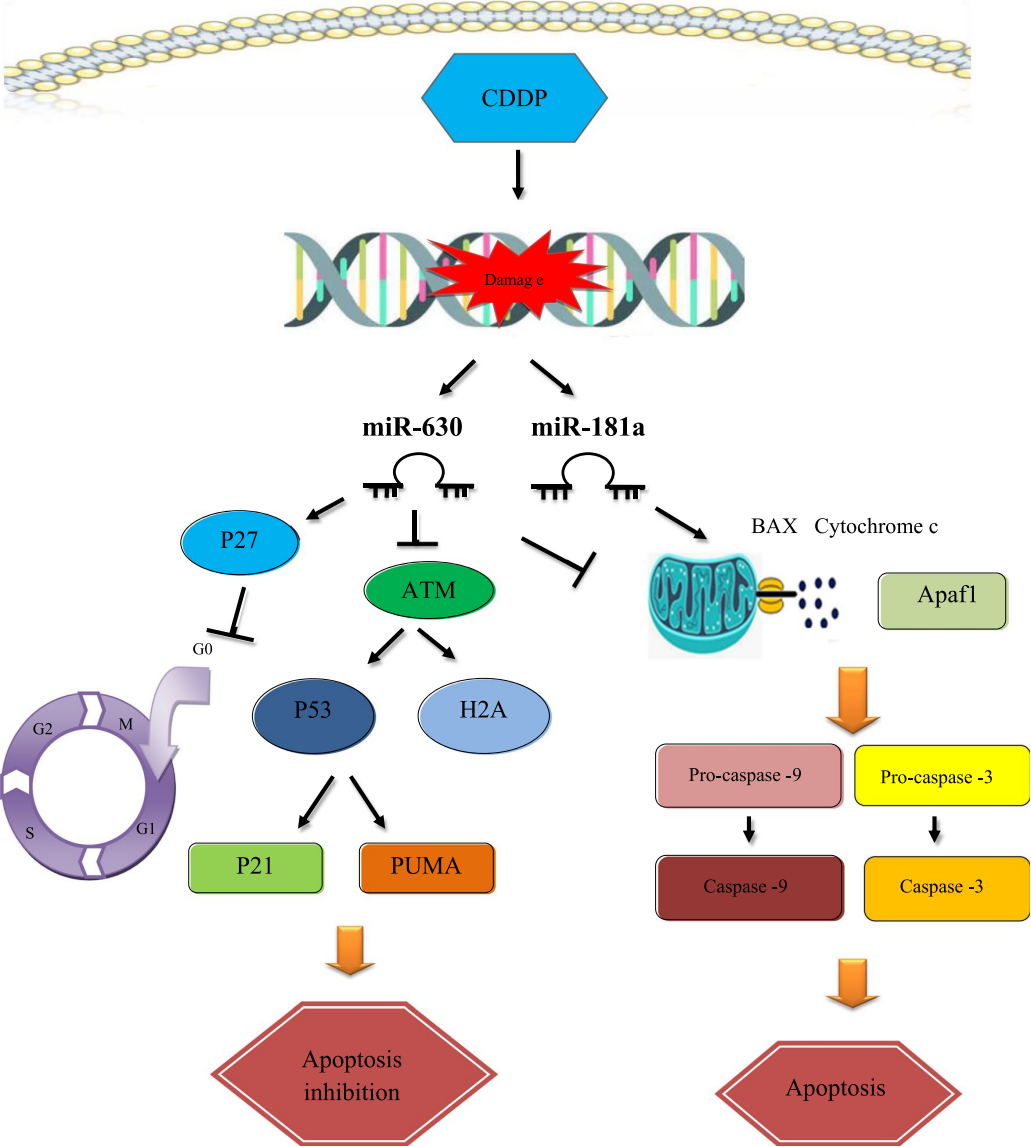
In non-small cell lung cancer (NSCLC) cells were treated with cisplatin (CDDP), miR-181a promoted cell death via BAX oligomerization and enhancement of procaspase-3 and procaspase-9 maturation, but miR-630 had an opposite effect. Also, miR-630 via blocking phosphorylation of ATM, H2AX, and P53 inhibited apoptosis. Moreover, miR-630 through enhancement of p27 expression, promoted cell cycle arrest in G0/G1 resulting in significant decrease in sensitivity to CDDP [40]

miR-630 has also been found to affect response of cancer cells to chemo/radiotherapy. Pre-miR-630 has been shown to decrease cisplatin (CDDP)-induced cell death in NSCLC cells. Pre-miR-630 could modulate several phase of the intrinsic pathway of apoptosis, such as oligomerization of BAX, dissipation of transmembrane potential in the mitochondria, and processing of caspases-9 and 3. Furthermore, pre-miR-630 has been found to obstruct early signs of the DNA damage responses, such as ATM (Ataxia Telangiectasia Mutated) phosphorylation (Fig. 2) [40].

In renal cancer cells, miR-630 up-regulation has been found to inhibit uptake of oxaliplatin by cancer cells through targeting organic cation transporter OCT2 (Organic Cation Transporter 2) [36]. On the other hand, expression of miR-630 has been demonstrated to be associated with higher response of colon cancer cells to radiotherapy. miR-630 has been shown to induce apoptosis in these cells following ionic radiation through targeting BCL2L2 (BCL2 Like 2) and TP53RK (TP53 Regulating Kinase). Moreover, CREB (CAMP Responsive Element Binding Protein 1) could regulate expression of miR-630, and demethylation could enhance expression of miR-630 [41]. In ovarian cancer cells, miR-630 inhibition could increase sensitivity to cisplatin [12], decline cell proliferation, invasion, and motility and improves apoptosis and sensitivity to paclitaxel [42]. miR-630 has also been shown to target IGF1R (Insulin Like Growth Factor 1 Receptor) to influence response of breast cancer cells to HER2-targeting agents [43]. Finally, in 4-aminobiphenyl-treated HCC cells, the level of miR-630 has been increased, leading to suppression of genes involved in DNA repair [44]. Table 3 shows the effect of miR-630 in the response of tumor cells to chemotherapy, radiotherapy and carcinogens.

**Table 3:** Effect of miR-630 in the response of tumor cells to chemotherapy, radiotherapy and carcinogens (*ANCTs* adjacent non-cancerous tissues)

Type of cancer	Expression pattern	Samples	Cell lines	Target	Related molecules and pathways	Function	Refs.
Renal cell carcinoma (RCC)	Up	15 pairs of RCC and ANCTs/mice	786-O, 769-P, HEK-293	OCT2	C-Myc	miR-630 is up-regulated by C-Myc and by suppression of OCT2 cause oxaliplatin resistance	[36]
Colorectal cancer (CRC)	–	–	Ls174T, SW480, HCT116, SW837, HR8348, and HT29	BCL2L2, TP53RK, caspase3, caspase6	CREB	High expression of miR-630 was positively associated with the radiosensitivity and apoptosis	[41]
Ovarian cancer (OC)	Up	53 cancerous, 25 benign, and 20 normal tissues	A2780 and SKOV3	PTEN	–	miR-630 inhibition could suppress proliferation, invasion, migration of cancer cells, and promote cell death and sensitivity to cisplatin	[12]
	Up	Mice	SKOV3-TR and SKOV3	APAF-1	–	miR-630 inhibition could decline proliferation, invasion, and motility of cells and improves apoptosis and sensitivity of cells to paclitaxel	[42]
Non-small cell lung cancer (NSCLC)	–	–	CL1-0,TL-4, A549, H1299, H23, H358	BCL2	–	A low level of miR-630 and a high level of BCL2 were correlated with poor response to cisplatin drug and colony formation	[34]
	Down	–	BEAS‐2B, NCI‐H1650, NCI‐H358, HCC827, NCI‐1299 and A549	AEG-1	CircMTDH.4	CircMTDH.4 via miR-630/AEG‐1 axis regulation could promote proliferation, invasion, metastasis, and resistance to chemotherapy and radiotherapy	[16]
	–	–	A549, H1650, H1975, HCC827, HCT116, HeLa	BAX,Caspase3, caspase9, ATM, P53, p-p53, H2AX, P27	P53 pathway, mitochondrial apoptotic pathway	miR-630 transfection to malignant cells could lead to low sensitivity to cisplatin	[40]
	–	Mice	PC9, PC9GR, CL97, H1975, H1650 and HCC827	YAP1, p-ERK, slug, Bad, and Bcl-2	ERK, SP1	In EGFR-mutated lung cancer cells, miR-630/YAP1/ERK axis may be responsible for TKI resistance	[37]
Lung cancer	–	–	A549 and H1299	–	E2F1, DROSHA	In cells treated with cisplatin, E2F1 via DROSHA enhancement could increase miR-630 expression	[45]
Prostate cancer (PCa)	–	–	PC-3, LNCaP, MDA-MB231, MCF-7, U2OS, and HeLaS3	–	–	Treatment of cells with gefitinib and luteolin could lead to growth suppression via miR-630 induction and GAK reduction	[46]
Pancreatic cancer (PC)	–	–	PANC-1 and MiaPaCa-2	IGF1R	–	Treatment of cells with 3-Cl-AHPC led to miR-630 over-expression which in turn reduced IGF1R and induced cell death	[47]
Hepatocellular carcinoma (HCC)	–	–	HepG2	RAD18	DNA repair pathway	In 4-aminobiphenyl-treated cells, the level of miR-630 was increased, leading to suppression of genes involved in DNA repair	[44]
Breast cancer (BC)	Down	GEO database	SKBR3, HCC1954 and MDA-MB-453	IGF1R, HER2, and EGFR	–	In drug resistant HER2-positive breast cancers, miR-630 could lead to greater effectiveness of chemotherapy drugs and reduced aggressive state of cells	[43]
Glioma	–	Mice	BT325, U373, U87 and U251	CDC14A	–	miR-630 inhibition via CDC14A up-regulation could promote cell proliferation and radioresistance	[48]

In lung cancer cells after DNA damage, miR-630 could inhibit proliferation and promote apoptosis by CDC7 (Cell Division Cycle 7) targeting, but so it could suppress apoptosis via other targets. In other words, miR-630 could have dual effect on apoptosis process and this issue goes back to its downstream targets [45].

**Fig. 3:**
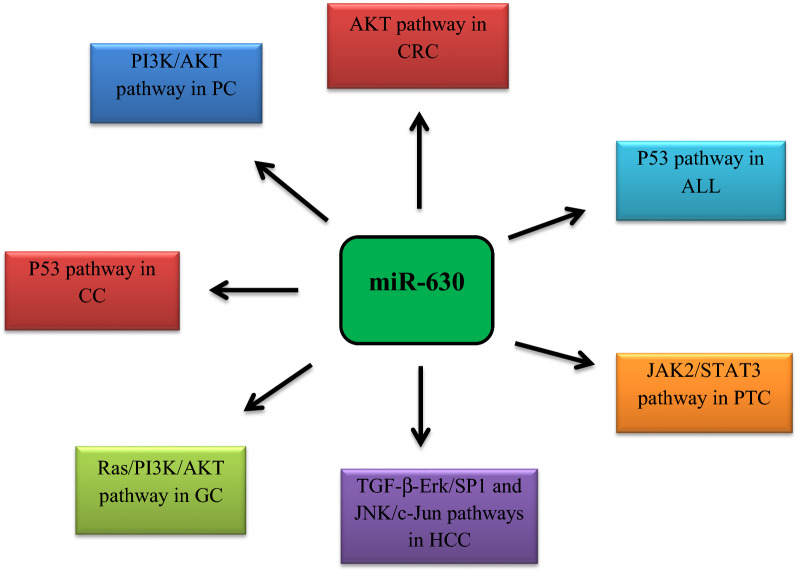
Participation of miR-630 in various signaling pathways in different malignancies. *PC* pancreatic cancer, *CRC* colorectal cancer, *ALL* acute lymphoblastic leukemia, *PTC* papillary thyroid carcinoma, *HCC* hepatocellular carcinoma, *GC* gastric cancer, *CC* cervical cancer

According to Rupaimoole et al. study, in ovarian and breast cancer cell lines, hypoxia condition led to miR-630 enhancement and targeting of Dicer by this miRNA [49]. This approach caused more tumor growth and metastasis. Moreover, the level of miR-630 expression affected the overall survival of patients, so that in patients who had a higher level of this miRNA, overall survival was more unfavorable [49]. The participation of miR-630 in various signaling pathways in different malignancies was shown in Fig. 3.

## miR-630 in non-malignant conditions

Expression of miR-630 has also been appraised in the context of non-neoplastic conditions (Table 4). For instance, this miRNA has been found to be up-regulated in human milk exosomes of HIV (Human Immunodeficiency Virus)-1 infected mothers compared with uninfected controls [50]. Differentially expressed miRNAs between these two groups have been enriched in pathways related with cell cycle transition, cancer, TGF-β pathway, FoxO pathway, fatty acid biosynthesis, p53 pathway and apoptosis [50]. Besides, the area under receiver operating characteristics curve of miR-630 has been measured to be 0.82, indicating its potential to detect HIV-1 infection in mothers [50]. In another study, miR-630 has been identified as a putative biomarker for prediction of AIDS (Acquired Immunodeficiency Syndrome) progression [51]. A high throughput method has shown down-regulation of miR-630 levels in palatal tonsils from IgA nephropathy patients compared with chronic tonsillitis. miR-630 has been shown to decrease expression of TLR4 (Toll-like Receptor 4), thus reducing levels of secreted IgA1 and increasing galactosylation of the IgA1 hinge region. Furthermore, TLR4 can influence expression levels of IL (Interleukin 1)-1β and IL-8 via NF-κB signaling to control both IgA1 levels and its glycosylation [52]. miR-630 has also been among miRNAs whose expressions have been correlated with the severity of nuclear opacity in age-related nuclear cataract [53]. This miRNA has been found to regulate expression of MAPK14 (Mitogen-Activated Protein Kinase 14) and a number of other genes [53].

**Table 4:** miR-630 in non-cancerous diseases (*ED* endothelial dysfunction; *NEF* normal endothelial function; *OB* obese)

Type of disease	Expression pattern	Samples	Cell lines	Downstream targets	Related molecules and pathways	Function	Refs.
AIDS	Up	27 HIV-1 positive women, 9 HIV-1 negative women	–	BCL2, BCL2L2, BCL2L11, and YAP1	–	miR-630 could be considered as a distinguishing biomarker between HIV1-infected mother milk and non-HIV1-infected mother milk	[50]
	Upper in chronic progressors than others	13 uninfected controls, 12 chronic progressors and 12 long-term nonprogressors (GEO dataset)	–	PELI1, LAPTM5, EXO1, ZNF131, TMED7	Enzyme linked receptor protein and receptor quanylyl cyclase signaling pathways	miR-630 could be considered as a putative biomarker for AIDS progression	[51]
IgA nephropathy (IgAN)	Down	27 tonsil tissues from IgAN patients and 20 tonsil tissues from patients with chronic tonsillitis	Tonsil mononuclear cell (TMC)	TLR4, IgA1	NF-kB signaling pathway	Over-expression of miR-630 caused TLR4 and IgA1 attenuation while glycosylation content of IgA1 was enhanced	[52]
Obstructive sleep apnea (OSA)	Down in ED	128 children: OB_NEF_ (n = 23), OB_ED_ (n = 20), OSA_NEF_ (n = 34), OSA_ED_ (n = 25), and Control (n = 26)/mice	Microvascular endothelial cells	416 different genes	10 various pathways as tight junction signaling pathways, NRF2-mediated oxidative stress responses and AMP kinase	miR-360 could be postulated as a risk factor of cardiovascular disease in OSA and obesity in children	[54]
Age-related nuclear cataract	Up	45 lens epithelium samples from cataract patients	HLE-B3	WEE2, MAP3K2, MAP4K3, STK39, DEFB132, MAPK14, ANKRD6, RAD1, ATG12, SGTB, MAP3K1, YES1, RB1CC1, BCL2L2, EDNRB, WEE1, FER, PAK7, DCLRE1C, UBXN2A, RAD18, PARP3, SLK, ATG2B, TMX3, BTBD3, TMX1, GPR37, CDC7, PPP2CA, IRAK3, TP63, RHD, FRK, CXCL13, POLR2D, GPX8, ITPR1, TLR4, SLK, UBE2N, SRPK2, CUL4B, TMX4, NBEAL1, CD226, CCL11, PIK3CA, DDB1, NLK	–	H_2_O_2_ treatment could overexpress miR-630 and correlated with nuclear opacity and progression of age-related nuclear cataract	[53]
Cataract	Up	Lens capsules from 25 cataract patients and 25 normal controls	SRA01/04	E2F3, Bax, cleaved caspase 3, Bcl-2, N-cadherin, vimentin, and a-SMA, E-cadherin	Axon guidance pathway	miR-630 in partnership with miR-378a-5p and by E2F3 targeting inhibited cell growth and EMT but induced cell death	[55]
Vitiligo	Down	Peripheral blood of 5 vitiligo patients and 5 non-vitiligo cases	–	–	–	miR-630 was identified as a differential expressed miRNA in vitiligo	[56]
Heterotopic ossification (HO)	Down	Sample of ectopic bone and serum from 146 HO cases, serums sample from 292 patients without HO after taking arthrolysis (control 1), and 292 patients with fracture healing (control 2)/mice	HD-MVEC, and 293T	Slug, osteocalcin, osteopontin and Runx2, VE-cadherin, occluding, N-cadherin, and vimentin	–	Suppression of endothelial-mesenchymal transition by miR-630 via targeting slug could be involved in the formation of ectopic bone in HO	[57]

## Discussion

miR-630 has different gene targets among them are those associated with cancer phenotype such as BCL2 [50], YAP-1 [27] and Slug [15]. Expression of this miRNA has been best assessed in the context of malignant conditions. However, the results of conducted studies in this field are contradictory. It is an oncogenic miRNA in renal cell carcinoma [8], multiple myeloma [24], colorectal cancer [9], acute lymphoblastic leukemia [10], ovarian cancer [11] and prostate cancer [13]. On the other hand, it is a putative tumor suppressor miRNA in lung [17], cervical [26], breast [28], thyroid [30] and esophageal tissues [31]. In a number of other tissues, data regarding the role of miR-630 in the carcinogenesis is conflicting [18, 19].

Circulating levels of miR-630 can be used as marker for separation of cancer patients from controls [22]. Moreover, expression profile of this miRNA has the potential to predict course of malignancy in several types of cancers (summarized in Table 2).

In addition, miR-630 can affect response of cancer cells to ionizing radiation, oxaliplatin and cisplatin. Thus, prior identification of miR-630 levels in tumoral tissues or circulation of patients might help in choosing the best efficient anticancer regimen in a personalized manner.

miR-630 is functionally linked with AKT, P53, TGFβ-ERK/SP1, JNK/c-Jun, PI3K/AKT and JAK2/STAT3 pathways which are among the mostly dysregulated pathways in cancers. Similar to other miRNAs, miR-630 has functional interactions with other classes of non-coding RNAs including long non-coding RNAs and circRNAs. H19, circMTDH.4 and circRNA_100269 are among transcripts whose interactions with miR-630 have been verified so far. High throughput evaluation of expression profiles of different classes of RNAs and additional functional analyses are needed for identification of other interacting molecules with miR-630.

## Conclusion

Cumulatively, miR-630 is involved in the pathoetiology of several malignant and non-malignant conditions. Yet, its role in the carcinogenesis is so complicated that it is not possible to assign a tumor suppressor or oncogene role for it in all tissue contexts. In spite of the presence of vast body of literature on the role of this miRNA in malignant conditions, few studies have addressed its contribution in non-malignant conditions. Among non-malignant conditions, the pathogenesis of IgA nephropathy, obstructive sleep apnea, age-related nuclear cataract, vitiligo and heterotopic ossification is related with levels of miR-630. Moreover, this miRNA has been found in exosome secreted in the human milk, possibly reflecting the disease status of the mother. This miRNA can be used as a potential biomarker for cancerous conditions. However, since its levels are different in diverse malignancies, it can be better used for patients’ follow-up rather than initial diagnosis. The presence of this miRNA in exosomes potentiates its applications in non-invasive diagnostic methods. However, future studies are necessary for validation of this hypothesis. A major limitation of studies that assessed the diagnostic or prognostic impact of miR-630 in human disorders is lack of validation in independent cohorts.

## Data Availability

The analyzed data sets generated during the study are available from the corresponding author on reasonable request.

## Abbreviations

miRNA: MicroRNA
circRNA: Circular RNA
ANCT: Adjacent non-cancerous tissue
NPC: Nasopharyngeal carcinoma
EMT: Epithelial–mesenchymal transition
RCC: Renal cell carcinoma
MM: Multiple myeloma
CRC: Colorectal cancer
ALL: Acute lymphoblastic leukemia
OC: Ovarian cancer
PCa: Prostate cancer
HCC: Hepatocellular carcinoma
PTC: Papillary thyroid carcinoma
NSCLC: Non-small cell lung cancer
GC: Gastric cancer
ESCC: Esophageal squamous cell carcinoma
CC: Cervical cancer
CDDP: Cisplatin
PC: Pancreatic cancer
BC: Breast cancer
IgAN: IgA nephropathy
HO: Heterotopic ossification
ED: Endothelial dysfunction
NEF: Normal endothelial function
OB: Obese
OSA: Obstructive sleep apnea
GEO: Gene expression omnibus
BCLC: Barcelona-clinic liver cancer
